# Reliability of the Spanish Version of the Movement Imagery Questionnaire-3 (MIQ-3) and Characteristics of Motor Imagery in Institutionalized Elderly People

**DOI:** 10.3390/jcm11206076

**Published:** 2022-10-14

**Authors:** Manuel Enrique Suárez Rozo, Sara Trapero-Asenjo, Daniel Pecos-Martín, Samuel Fernández-Carnero, Tomás Gallego-Izquierdo, José Jesús Jiménez Rejano, Susana Nunez-Nagy

**Affiliations:** 1 Amavir Torrejón de Ardoz Nursing Home, 28850 Torrejón de Ardóz, Spain; 2 Department of Nursing and Physiotherapy, Faculty of Medicine and Health Sciences, University of Alcalá, 28805 Alcalá de Henares, Spain; 3 Humanization in the Intervention of Physiotherapy for the Integral Attention to the People (HIPATIA) Research Group, Physiotherapy Department, Faculty of Medicine and Health Sciences, University of Alcalá, 28801 Alcalá de Henares, Spain; 4 Physiotherapy and Pain Group, Physiotherapy Department, University of Alcalá, 28871 Alcalá de Henares, Spain; 5 Department of Physiotherapy, University of Seville, 41009 Seville, Spain

**Keywords:** physical therapy, imagery, institutionalized persons

## Abstract

Motor imagery (MI) training is increasingly used to improve the performance of specific motor skills. The Movement Imagery Questionnaire-3 (MIQ-3) is an instrument for assessing MI ability validated in Spanish although its reliability has not yet been studied in the elderly population. The main objective of this study was to test its reliability in institutionalized elderly people. Secondarily, we studied whether there are differences according to gender and age in MI ability (measured by the MIQ-3) and in temporal congruency (measured by mental chronometry of elbow and knee flexion-extension and getting up and sitting down from chair movements). The subjects were 60 elderly, institutionalized, Spanish-speaking individuals without cognitive impairment or dementia, and aged between 70 and 100 years. Cronbach’s alpha showed high internal consistency in the internal visual and external visual subscales and moderate in the kinesthetic subscale. The intraclass correlation coefficient showed good test-retest reliability for all three subscales. Mixed factorial analysis of variances (ANOVAs) showed that MI ability decreased with increasing age range, the imagery time decreased concerning the execution of the same movement, and there were no gender differences in either IM ability or temporal congruence. The Spanish version of the MIQ-3 is a reliable instrument for measuring MI ability in institutionalized elderly.

## 1. Introduction

Motor imagery (MI) comprises imagining a movement without executing it to optimize motor skills [1]. It is a specific cognitive process in which the planning of a movement is carried out without executing it through actual physical movement [2], and it is observed to have the same components and involve the same brain areas as when a real movement is performed [2,3]. This process can also be explained thanks to the existence of the psychoneuromuscular theory, whose foundations support the idea that MI improves motor learning based on the role played by mirror neurons when these are activated during the visualization of a movement in mental practice [2]. In turn, the motor schema involved in the actual activity is reinforced during MI so that the processes occurring during imagery aid performance, reinforcing coordination patterns for motor skill development [2].

Therefore, MI practice is a technique that is increasingly used in the therapeutic context to improve the performance of specific motor skills, and whenever possible, it is combined with physical practice [2,4,5]. Thus, MI practice has been studied in healthy subjects, athletes [6,7,8,9,10,11,12], as well as in multiple neurological conditions [13,14,15,16] and pain conditions [17,18,19,20], among others. It has also been used in combination with virtual reality using brain–computer-interface-based systems in people with neurological sequelae [20,21].

A recent systematic review showed improved balance, mobility, and gait speed among the therapeutic benefits of MI training in older people without neurological conditions [22]. For an MI training program to be effective, the ability to generate imagery needs to be assessed [23]. However, there are few studies on MI capacity in the elderly, specifically in institutionalized elderly people. As is well-known, the institutionalization of elderly people in nursing homes is one of the best options when they can no longer live at home. This change entails social, affective, self-esteem, and motivation losses, increasing hopelessness about old age and suffering from chronic diseases and/or disabilities [24]. Among the latter are those caused by injuries to the locomotor system, as most institutionalized older people are below average in terms of lower- and upper-limb muscle strength, which is associated with a low level of physical activity [24]. High physical activity levels have been associated with a greater capacity to generate motor mental images [20], and MI capacity must be trained in older people to obtain positive results [22].

Imagery capacity can be assessed in different ways. Studies on the elderly have pointed out that MI capacity should be carefully assessed, where MI capacity questionnaires and mental chronometry would be very appropriate, among others [25]. Thus, MI can be assessed in terms of vividness through self-reported questionnaires such as the Movement Imagery Questionnaire-3 (MIQ-3) and temporal characteristics through temporal congruency through mental chronometry. Both forms of MI assessment are complementary, as each assesses different aspects of MI.

The MIQ-3 is an instrument validated in Spanish, consisting of 12 items grouped into three subscales. It is a multidimensional measure that has been used to measure the capacity for internal, external, and kinesthetic imagery and whose psychometric properties have shown good internal consistency as well as internal reliability and predictive validity, suggesting that it is a suitable instrument for assessing MI abilities in healthy and young people of both sexes [26,27]. It is important to consider the age of the subject, as it has been shown that the capacity for imagery decreases progressively with age, affecting the development of motor skills [22]. Furthermore, scientific evidence suggests that the MI capacity of some movements is modified due to some age-related alterations, indicating that aging produces selective effects on mental imagery [28]. Nevertheless, the reliability of the MIQ-3 for use in the elderly has not been tested so far, nor have similar questionnaires been validated for use in the elderly. A recent systematic review of MI assessments suggests that more studies are needed in this context, including older populations [29].

On the other hand, temporal congruence is considered the time course of mental operations between simulated and real movements [25]. It is measured through mental chronometry, measuring the time it takes the subject to execute a movement and the time it takes to imagine that movement.

Liu et al. [30] compared MI ability among populations distributed by gender and in three age ranges. They concluded that temporal congruency is preserved with age for simple and usual movements and is impaired for limited and unusual movements. They also observed a lower capacity for internal visual and kinesthetic imagery in people over 60 years of age relative to younger people. Regarding gender, MI ability was found to be better in men than in women. However, some studies have found no significant gender differences in this population [31]. Another study found that women may overestimate the imagined task relative to actual practice, while men underestimate it [32].

Therefore, more studies are needed to support the use of the MIQ-3 and mental chronometry to assess MI ability for these groups of elderly people, paying attention to differences according to age and gender.

This study’s main objective was to determine whether the Spanish version of the MIQ-3 is a reliable instrument for measuring motor imagery ability in institutionalized elderly people. The secondary objectives were to explore MI ability as a function of this population’s age range, gender, and temporal characteristics (through temporal congruence). As hypotheses, it was established that the Spanish version of the MIQ-3 is a reliable instrument to measure MI capacity in this population and that MI capacity measured by this questionnaire is higher in males than in females and decreases as the age range increases. It is expected that temporal congruency is better preserved in males than in females, and it similarly decreases with age.

## 2. Materials and Methods

The design adopted corresponded to reliability studies. A repeated-measures cross-sectional design was carried out on the subjects in the sample. In addition, the recommendations established in the Guidelines for Reporting Reliability and Agreement Studies (GRRAS) [33] were followed.

### 2.1. Participants

The study sample comprised 60 institutionalized elderly people: 27 men (45%) and 33 women (55%). The 60 subjects were divided into groups according to three age ranges. The first group consisted of 16 people (26.67%) aged between 70 and 79 years (mean (M) = 72.6; standard deviation (SD) = 1.86). The second group consisted of 26 persons (43.33%) aged 80–89 years (M = 84; SD = 1.92), and the third group consisted of 18 persons (30%) aged 90–100 years (M = 92.5; SD = 2.0) (Table 1).

The inclusion criteria for the study were: Spanish-speaking, aged 70–100 years, of both genders, and without cognitive impairment or dementia as measured by Pfeiffer’s Short-Portable Mental State Questionnaire (SPMSQ) [34,35] and Yesavage’s Geriatric Depression Scale [36]. The exclusion criteria were having suffered traumatic processes in the last 6 months and being under treatment with central nervous system suppressant drugs. Participants were recruited from the “Residencia de mayores Amavir” social-health center in Torrejón de Ardoz after the center’s medical committee granted permission. Participation was voluntary after signing the informed consent form.

### 2.2. Data Collection Instrument

The MIQ-3 is composed of 12 items grouped into three subscales (internal visual imagery, external visual imagery, and kinesthetic imagery), which allow for the assessment of MI in both genders about four movements involving knee elevation, jumping, arm movement, and leaning forward at the waist, all repeated in three subscales [26,27]. These movements are described in each statement to be performed under instructions that indicate the initial position, the action, the mental task, and the score using a seven-point Likert scale, indicating the difficulty or ease of “seeing” and “feeling” the movements [26]. It has been validated in different languages and different populations [27].

### 2.3. Variables

Gender and age were considered independent and controlled sociodemographic variables in the study. In addition, MI, measured through the MIQ-3, and temporal congruence, measured through mental chronometry, were considered dependent variables. Three movements were performed to measure mental chronometry: elbow flexion-extension, knee flexion-extension, and getting up and sitting down from a chair.

### 2.4. Procedure

The same researcher oversaw carrying out the two data collection sessions. To homogenize the conditions, the verbal orders given to the subjects during the sessions were standardized before the sessions and carried out in the same room and under the same environmental conditions.

In the first session, the MIQ-3 was administered, and time congruency was measured by mental chronometry of elbow flexion-extension, knee flexion-extension, and getting up and sitting down from a chair. Before performing the mental chronometry task, the experimenter gave a physical demonstration of the movements to be performed. Afterward, using previously standardized commands, they were asked to perform the different movements and then try to imagine them. The execution and imagination times were calculated employing a stopwatch, which was pressed by the researcher at the subjects’ “start” and “stop” commands at the moments of both the actual execution of the movements and the imagined execution. Each movement was performed and imagined on three occasions, and each movement’s mean mental chronometry value was then calculated.

In the second session (after one week), the MIQ-3 was administered again for the study of retest reliability.

### 2.5. Statistical Analysis

Statistical analysis was carried out using SPSS, version 26.0 for Windows (International Business Machines Corporation (IBM), Armonk, NY, USA).

First, the descriptive analysis of the results obtained in the two measurements made with the questionnaire (test and retest) was carried out as well as the mean and the difference between the measurements.

Subsequently, internal consistency was assessed by calculating Cronbach’s alpha coefficient. Interpretation was based on the following values: very low (0 to 0.2); low (0.2 to 0.4); moderate (0.4 to 0.6); good (0.6 to 0.8); and high (0.8 to 1). Adequate internal consistency was between 0.7 and 0.939 since excessively high values could indicate redundant items within the questionnaire [37].

The test-retest reliability of each questionnaire item was analyzed by calculating the value of the weighted kappa coefficient, following Cicchetti’s method. The weighted kappa coefficient values were interpreted following the classification established by Landis and Koch [38]. Agreement was no agreement if the Kappa index took a value of 0.00; negligible if it was between 0.01 and 0.20; medium if it was between 0.21 and 0.40; moderate between 0.41 and 0.60; substantial between 0.61 and 0.80; and near perfect between 0.81 and 1.00 [39,40]. These analyses were carried out with the statistical program Epidat 4.2. (Consellería de Sanidade, Xunta de Galicia, Spain; Pan American Health Organization (PAHO); CES University, Medellin, Colombia).

The test-retest reliability of each subscale was analyzed by calculating the intraclass correlation coefficient (ICC) using a two-factor model with mixed effects and absolute agreement. The 95% confidence interval for the ICC values was also calculated. The Weir criteria [41] were followed to interpret the ICC values, where values of 0.50 to 0.69 are considered moderate, values of 0.70 to 0.89 as high, and values of 0.90 and above as excellent.

The analysis of differences in MI ability measured by the MIQ-3 was carried out according to sex and age considering that the sample was distributed into three age groups. Two mixed factorial analysis of variances (ANOVAs) were used for this purpose. This design was used to determine whether the differences analyzed were because of the inter-subject factor (either sex or age range). In this sense, in the first mixed factorial analysis of variance (ANOVA), the inter-subject factor was the sex of the subjects, while in the second one, the age range was considered. The hypothesis of interest was the inter-subject factor interaction by time, with an a priori alpha level of 0.05. In addition, the effect size of the observed differences was estimated by calculating the partial eta-squared coefficient (η_p_^2^). The assumption of the sphericity hypothesis was tested using Mauchly’s test. In those cases where the assumption of sphericity was not met, the Greenhouse–Geisser correction was used. In addition, the analysis was completed by employing multiple comparison tests, using the Bonferroni correction, and determining the effect size, and Cohen’s d was calculated.

The data analysis for time congruence was carried out using a mixed factorial ANOVA with respect to sex and age. For differences that conformed to the normal and were homoscedastic, the Mann–Whitney U test was used for those differences that did not conform to the normal, and the effect size was determined by calculating Rosenthal’s r with the formula: r = Z/√N [42,43]. Kruskal–Wallis ANOVA was performed for comparison according to age range.

## 3. Results

### 3.1. Descriptive Analysis

The descriptive analysis of the scores obtained in each subscale of the MIQ-3 showed that in the second session, the values in the three subscales were higher than those obtained in the first session. In this regard, the differences obtained between the means between the two sessions were −2.50 in the external visual subscale, followed by −2.25 in the internal visual subscale and −2.00 in the kinesthetic scale (Table 2).

### 3.2. Analysis of Internal Consistency

The Cronbach’s alpha analysis showed values that allowed us to establish a high internal consistency in the case of the questionnaire. The internal and external visual subscales showed good internal consistency, while the kinesthetic subscale showed moderate consistency (Table 3).

### 3.3. Analysis of the Test-Retest Reliability

**Figure 1:**
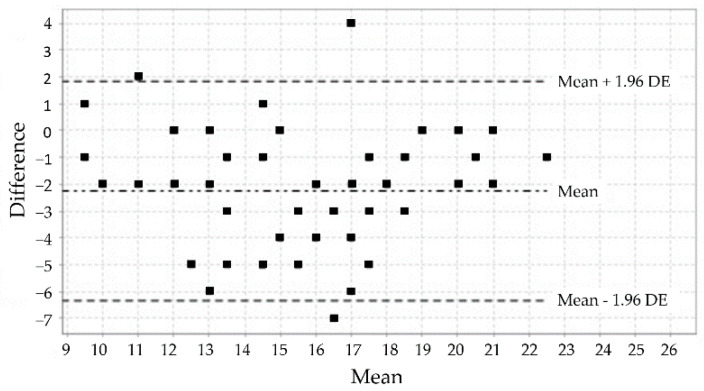
Bland–Altman plot of the internal visual subscale of the MIQ-3.

**Figure 2:**
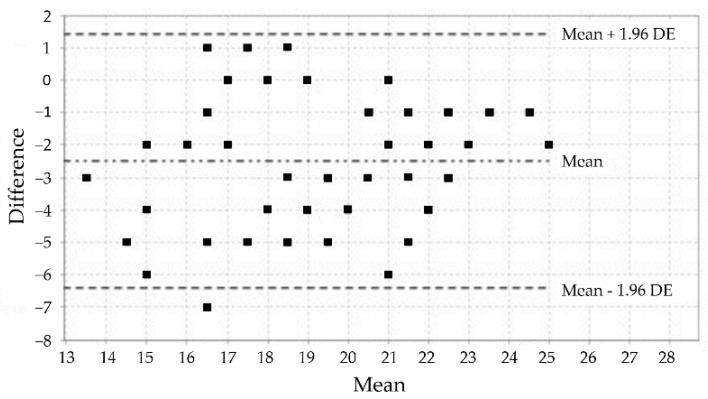
Bland–Altman plot of the external visual subscale of the MIQ-3.

**Figure 3:**
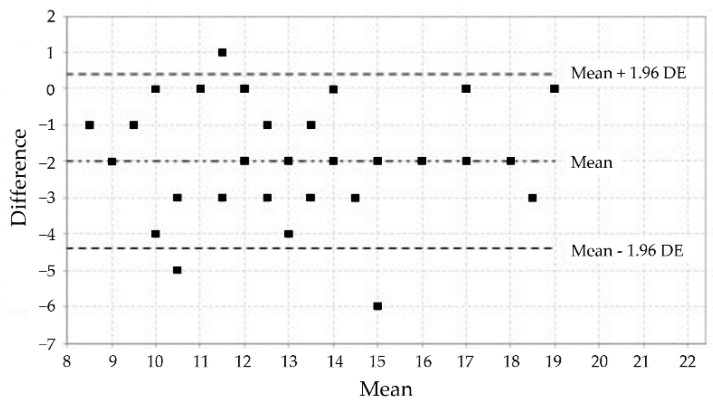
Bland–Altman plot of the kinesthetic subscale of the MIQ-3.

The analysis using the weighted kappa coefficient established that, of the 12 items, 8 showed a medium degree of agreement, 1 item showed a moderate degree of agreement, and 3 items showed substantial agreement (Table 3). The analysis corresponding to the test-retest reliability of each subscale by calculating the ICC made it possible to establish good reliability values (Table 3). These results are confirmed by the visual distributions of the Bland–Altman plots for the test-retest comparison of the three subscales of the MIQ-3 (Figure 1, Figure 2 and Figure 3).

### 3.4. Analysis of Differences in MI Ability as Measured by the MIQ-3 Concerning Sex and Age

The mixed factorial ANOVA indicated in the case of the comparison according to sex in the three subscales, i.e., internal visual, external visual, and kinesthetic, that there was no significant interaction between the within-subjects factor (the two measurements taken) and the between-subjects factor (sex). There was also no significant effect of the inter-subject factor, but there was a significant effect of the intra-subject factor. Both sexes behaved similarly, with significantly higher values in the second session than in the first session. There were no differences between males and females in either measurement (Table 4).

Regarding the differences according to MI age range (MIQ-3) in the three subscales, namely internal visual, external visual, and kinesthetic, it was found that there was a significant interaction between the within-subjects factor (the two measurements taken) and the between-subjects factor (age range). There was also a significant effect of the inter-subject and intra-subject factors. The three age ranges behaved similarly in the three subscales (internal visual, external, and kinesthetic), with significantly higher values in the second session compared to the first (Table 5).

On the other hand, there were statistically significant differences between the values of the three age ranges in the internal visual and kinesthetic subscales, with the 70–79 age group presenting the highest values, followed by the 80–89 age group and, finally, the 90–100 age group with the lowest values. In the external visual subscale, the group aged 70 to 79 years presented the highest values, followed by the group aged 80 to 89 years and, finally, the group aged 90 to 100 years with the lowest values, with there being significant differences between the group aged 70 to 79 years and the other two groups (80 to 89 and 90 to 100 years). However, the differences observed were not significant between the 80–89 and 90–100 age groups (Table 5).

### 3.5. Analysis of Temporal Congruence Concerning Sex and Age

Mixed factorial ANOVA was performed to compare, according to sex, the three movements corresponding to temporal congruency: elbow flexion-extension, knee flexion-extension, and getting up and sitting down on the chair. The results obtained indicated that in the case of the first two movements (elbow and knee flexion-extension), there was no significant interaction between the intra-subject factor in the two measurements (performed and imagined) and the inter-subject factor (sex). There was also no significant effect of the intra-subject factor, but there was a significant effect of the inter-subject factor, F_(1, 58)_ = 5.48; *p* = 0.023; η_p_^2^ = 0.086 in the elbow flexion-extension movement and F_(1, 58)_ = 10.06; *p* = 0.002; η_p_^2^ = 0.148 in the knee flexo-extension movement. Both sexes behaved differently. In men, the values of the executed measurement (elbow flexion-extension M = 3.71, SD = 0.42; knee flexion-extension M = 4.06, SD = 0.41) were higher than those of the imagined measurement in both subscales (elbow flexion-extension M = 3.64, SD= 0.33; knee flexion-extension M = 3.90, SD = 0.38). In women, the values of the executed measurement (M = 3.96, SD = 0.48) were slightly higher than those of the imagined measurement in the elbow movement (M = 3.94, SD = 0.60), and the values of the executed measurement (M = 4.06, SD = 0.41) were slightly lower than the imagined one in the knee movement. In both movements, differences (*p* < 0.05) were found between men and women in both the executed and imagined measurements, with women’s values being significantly higher. In the intra-group comparison of the two movements of the two measurements carried out (the executed and the imagined), it was found that neither in men nor in women were there significant differences between the two measurements.

As for the get up and sit down on the chair movement, this mixed factorial ANOVA showed no significant interaction between the intra-subject and inter-subject (sex) factors, but there was a significant effect of the inter-subject factor F_(1, 58)_ = 6.72; *p* = 0.012; η_p_^2^ = 0.104 and the intra-subject factor F_(1, 58)_ = 54.23; *p* < 0.001; η_p_^2^ = 0.483. Both sexes behaved similarly, with higher values for the imagined measurement (male M = 5.10, SD = 0.71; female M = 5.47, SD = 0.60) than for the executed measurement (male M = 4.61, SD = 0.41; female M = 4.93, SD = 0.55). There were statistically significant differences (*p* < 0.05) between men and women in the two measurements, with women having significantly higher values. In the intra-group comparison of the two measurements carried out, we obtained that in both men and women, there were significant differences (*p* < 0.05) between the two measurements, with the imagined scores being significantly higher.

Next, the results obtained for temporal congruence were analyzed, i.e., the difference in the three movements of elbow flexion-extension, knee flexion-extension, and getting up and sitting down on the chair, comparing the two measurements (executed less imagined) concerning sex. The Mann–Whitney U-test was used for all movements. These analyses showed no gender differences in the three movements (Table 6).

Mixed factorial ANOVA was performed to analyze, according to age range (inter-subject factor), the two measurements (performed and imagined) of the three movements corresponding to temporal congruency: elbow flexion-extension, knee flexion-extension, and getting up and sitting down on the chair. These analyses showed a significant interaction between movement execution and imagery (intra-subject factor) and age range (inter-subject factor) in the elbow flexion-extension (F_(2, 57)_ = 9.68, *p* < 0.001; η_p_^2^ = 0.253) and knee flexion-extension (F_(2, 57)_ = 5.97, *p* = 0.004; η_p_^2^ = 0.173). There was also a significant effect of the inter-subject factor (elbow flexion-extension F_(2, 57)_ = 10.36, *p* < 0.001; η_p_^2^ = 0.267; knee flexion-extension F_(2, 57)_ = 6.42, *p* = 0.003; η_p_^2^ = 0.184) but no significant effect of the intra-subject factor. Thus, the three age ranges did not behave similarly in both the elbow flexion-extension movement and the knee flexion-extension movement. While the values of the imagined measurement decreased compared to the executed one in the 80–89 years and 90–100 years age groups, the values increased in the 70–79 years age group. In both movements, there were significant differences (*p* < 0.05) between the value of the executed and imagined measurement in the 70–79 years (the value of the imagined measurement being higher) and 90–100 years (the value of the executed measurement being higher in this case), while in the 80–89 years group, there were no differences between the two measurements. On the other hand, in the executed measurement in both the elbow flexion-extension and knee flexion-extension movements, there were statistically significant differences (*p* < 0.05) between the three age ranges, with the 70–79 age group showing the lowest values, followed by the 80–89 age group and, finally, the 90–100 age group showing the highest values. However, in the imagined measurement of the elbow flexion-extension movement, there were only significant differences (*p* < 0.05) between the 70–79 years and 90–100 years groups, with no significant differences between the other groups, and in the knee flexion-extension movement, there were no significant differences between any of the three age ranges.

Finally, in the movement of getting up and sitting down from a chair, there was a significant effect of the intra-subject factor F_(1, 57)_ = 64.25, *p* < 0.001; η_p_^2^ = 0.530 (the two measurements taken, executed, and imagined) and inter-subject factor F_(2, 57)_ = 20.47, *p* < 0.001; η_p_^2^ = 0.418 (the three age groups considered), but there was no significant interaction between the two factors. In the three age ranges, in this movement of standing up and sitting down, there were significant differences (*p* < 0.05) between the executed and imagined measurement, with higher values for the imagined measurement. On the other hand, in the executed measurement, there were significant differences (*p* < 0.05) between the three age ranges, with the 90–100 age group showing the highest values, followed by the 80–89 age group and, finally, the 70–79 age group with the lowest values of the three. Meanwhile, in the imagined measurement, there were only significant differences between the 70–79 age group and the 90–100 age group.

Next, the results obtained for temporal congruence were analyzed, i.e., the difference in the three movements of elbow flexion-extension, knee flexion-extension, and getting up and sitting down on a chair, comparing the two measurements (performed and imagined) according to age range by carrying out a Kruskal–Wallis ANOVA. There were no differences between the three age ranges compared in temporal congruence in standing and sitting. However, there were significant differences (*p* < 0.05) between the three age ranges in the temporal congruence in elbow flexion-extension and knee flexion-extension movements. Specifically, in elbow flexion-extension, there were significant differences (*p* < 0.05) between the 70–79 age group and the other two groups. In the knee flexion-extension movement, there were significant differences (*p* < 0.05) between the subjects aged 70–79 years and the group aged 90–100 years (Table 7).

## 4. Discussion

The aim of this study was to test the reliability of the Spanish version of the Movement Imagery Questionnaire-3 in 60 institutionalized elderly people. The first translation, cultural adaptation, and validation of the Spanish version of the MIQ-3 [27] have recently been published. This work is focused on healthy young people. For older people, no work has been found that evaluates the reliability of this test or similar questionnaires for older people.

The descriptive analysis of the results obtained in the two measurements made with the questionnaire (test and retest) showed higher values in the second session in the three subscales, which suggests that the participants showed a better ability to imagine measured with the MIQ-3 the second time they took the questionnaire. This could be the result of the MI practice implicit in the development of the first session, in which subjects performed both the questionnaire itself and the imagery tasks related to temporal congruence. This is consistent with the results of the study by Rufino et al. [44], where it was observed that a single MI session already induces use-dependent brain plasticity.

Cronbach’s alpha was acceptable for all the values obtained, and none of the items was redundant [37]. In this sense, these results are consistent with Trapero-Asenjo et al. [27] even though the value obtained was lower than in the study by the authors.

Concerning the subscales, the analysis revealed a good internal consistency for both the internal visual subscale (0.615) and the external visual subscale (0.651) and a moderate internal consistency (0.556) for the kinesthetic subscale. These results show that the values obtained were lower than those obtained in the study by Trapero-Asenjo et al. [27], which indicated a high internal consistency for the three subscales, with 0.849 for the internal visual subscale, 0.837 for the external visual subscale, and 0.615 for the kinesthetic subscale. These lower values in the study by Trapero-Asenjo et al. [27] coincide with the lower values obtained in validating the MIQ-3 in Portuguese by Mendes et al. [8].

The test-retest reliability analysis of each of the 12 items that make up the questionnaire by means of the weighted and interpreted kappa coefficient value showed medium, moderate, and substantial degrees of agreement in 8 items. Therefore, these correspond to adequate test-retest reliability values as established in the classification of Landis and Koch in 1977 [38]. These findings partially coincide with the results obtained by Trapero-Asenjo et al. [27], who found moderate to substantial agreement on all 12 items. In contrast, in the present investigation, items 1 and 3 of the internal visual subscale; items 1, 2, and 3 of the external visual subscale; and items 1, 2, and 3 of the kinesthetic subscale showed a medium degree of agreement, i.e., below the values obtained by Trapero-Asenjo et al. [27].

The results of the test-retest reliability analysis of each MIQ-3 subscale by calculating the ICC showed for the external visual subscale an ICC of 0.534 (95% confidence interval (CI) = 0.07, 0.80; *p* < 0.001), in the internal visual subscale a CCI of 0.611 (95% CI = 0.02, 0.83; *p* < 0.001), and the highest value was in the kinesthetic subscale, with a CCI of 0.691 (95% CI = 0.07, 0.90; *p* < 0.001). These values were interpreted according to Weir’s criteria [41], showing internal consistency with moderate values in all subscales. These findings are consistent with the results presented by Trapero-Asenjo et al. [27]; however, the values obtained were lower, as the ICC of the three scales were high in the study by Trapero-Asenjo et al. [27], while in this study, the values were moderate.

All these results suggest that the Spanish version of the MIQ-3 is a reliable measure of MI capacity for use in institutionalized elderly people. The study by Suica et al. [29] showed that the questionnaires for assessing MI with the best psychometric properties were the Movement Imagery Questionnaire (MIQ) [45] as well as its versions Movement Imagery Questionnaire—Revised (MIQ-R) [46], MIQ-3, and the Vividness of Movement Imagery Questionnaire (VMIQ-2) [47]. The same study showed that most studies assessing MI had been conducted in a young population [29], thus highlighting the need to validate MI assessment tools in the elderly. On the other hand, of all these questionnaires, only the MIQ-3 and the VMIQ-2 assess MI ability and MI vividness, respectively, in the three subscales of internal visual, external visual, and kinesthetic imagery [26,47]. It has been shown that these three forms of imagery are separate but related constructs [26,46], so the assessment of all three is of particular importance for both research and clinical applicability. Thus, this is the first study to confirm that MI capacity can be reliably assessed in institutionalized elderly people using the Spanish version of the MIQ-3, which currently represents the most suitable questionnaire for assessing MI ability on all three subscales. In clinical applicability, it has been proven that the ability to image can be improved with practice [48]. The results of this study will allow the design of more effective MI programs in the elderly since they not only allow the evaluation of MI through these questionnaires at the beginning of programs with MI but also allow them to monitor changes in the capacity of MI that are happening along the program.

On the other hand, studies have been carried out in which the capacity and vividness of MI in elderly people has been explored through questionnaires and their temporal characteristics through temporal congruence studies. It has been seen that the study of both issues is important, as it was pointed out that the ability to imagine and the temporal congruence are separate constructs and should be evaluated separately because they are affected differently by age [32]. To explore those questions, the present study analyzed the scores of the three subscales of MIQ-3 and three tasks of time congruence according to sex and age in the elderly population.

Thus, regarding the secondary objectives, in the analysis of differences in MI ability measured through the MIQ-3, there were no differences between men and women in the two measurements. These results partially coincide with the findings reported by Campos et al. [31]. They assessed in a sample of adult subjects whether there are age and gender differences by using self-report and a performance-based test, reporting no significant differences between the sexes concerning MI ability even though males scored higher than females.

Regarding the differences according to MI age range (MIQ-3), the analysis of the results suggests that the three age ranges behaved similarly in the three subscales (internal visual, external, and kinesthetic), with significantly higher values in the second session compared to the first. This could be associated with a learning process derived from the execution of the movement.

On the other hand, there were statistically significant differences between the values of the three age ranges in the internal visual and kinesthetic subscales, with the group of septuagenarians presenting the highest values, followed by the group of octogenarians and, finally, the group of nonagenarians and centenarians with the lowest values. In the external visual subscale, the decrease in scores with increasing age was similar to the other two subscales. However, in this case, the significant differences were between the septuagenarian group with respect to octogenarians and nonagenarians to centenarians, but the differences observed were not significant between these last two groups. These results confirm the findings of Subirats et al. [32]. They found that MI ability (measured with the Vividness of Movement Imagery Questionnaire-2 and MI timing using the performances of the real Timed Up and Go (rTUG) test) is affected by age, with a tendency for MI to decrease with age in the present study, with no significant differences between the group of nonagenarians and centenarians with respect to the group of adults aged 70–79 years. They also confirm the results that Mulder et al. [49] obtained, which showed that older participants had slightly worse MI ability (measured with the Vividness of Movement Imagery Questionnaire) than younger participants.

Similarly, they corroborate the findings of Schott [50], whose study examined key characteristics of MI ability in three groups of healthy older men and women (measured with the Movement Imagery Questionnaire, the Controllability of Motor Imagery test, and two different chronometry tests) distributed across three age groups (60–69, 70–79, and ≥80 years) and 40 younger subjects aged 20–30 years. They found that MI ability was better in younger adults compared to older adults aged 70 years and older but not in older adults aged 60–69 years. However, as noted above, IM ability can be improved with practice, and low scores are not exclusive to IM programs [48].

Regarding the differences in temporal congruence, no significant differences were observed concerning the differences between gender. However, there were differences in the time used to perform and imagine the movements. Thus, both sexes took significantly longer to imagine than to execute the movement of sitting down and getting up from the chair, and on the other hand, the men took longer to execute than to imagine the less global movements of flexion-extension of the elbow and knee, whereas the women had very similar results in both moments of the task. Therefore, it seems that in the simplest movements, the imagery of the movement tends to have a shorter duration than the movement itself, while in more global movements, such as getting up and sitting down, the imagery of the movement is reproduced more slowly than the execution of the same movement in the elderly population. The previous study by Saimpont et al. [25] pointed out that temporal congruence in the elderly seems to be more reserved in simple and usual movements, so all these issues should be further explored in future studies.

Regarding the differences in temporal congruence with respect to age, differences were only observed in the elbow and knee flexo-extension movements. In both cases, it was seen that as age increased, the imagery time was significantly reduced compared to the execution time of the same movement. This again suggests that the ability to maintain temporal congruence varies with increasing age. In this sense, Schott et al. [50] observed that from the age of 79, the difference between the values of imagery and execution of the movement increased progressively, so this issue should be further explored in future studies.

### Limitations

To conclude, for the analysis of the study’s main objective, the sample of five people per item was adequate [51], and for the study of the characteristics of the imagery, the total sample was also adequate, with a size equal to 60 subjects. However, as a limitation of the study, the sample consisted of 26 participants in the 80–89 age group, 18 subjects in the 90–100 age group, and 16 subjects in the 70–79 age group. It would be desirable to carry out studies with a larger sample in each age range to obtain more representative results for each age group and validate the results in non-institutionalized elderly people.

In the study, the sample was selected based on the absence of cognitive impairment or dementia as well as depressive disorders, traumatic processes in the last 6 months, and treatment with central nervous system suppressant drugs. Thus far, no studies have explored how other health aspects may influence the ability to imagine in older people (high blood pressure, diabetes, vision, and hearing problems, among others). It is suggested that these data could be collected in future studies. Although it is not possible to establish relationships due to the design, this will help to understand the sample’s characteristics better.

Finally, it should also be considered that the participants only belong to one center, so the results should not be extrapolated. Therefore, future studies should include larger samples and institutionalized and non-institutionalized elderly in different institutions.

## 5. Conclusions

The study allows us to conclude that the Spanish version of the MIQ-3 is a reliable instrument for measuring MI capacity in institutionalized elderly people.

The findings obtained did not demonstrate significant differences in MI ability measured with the MIQ-3 between women and men in this population. However, the results of this study support the hypothesis that MI ability decreases with the increasing age range.

In relation to temporal congruency, the analyses did not show differences between genders and observed that as age increases, the imagery time decreases with respect to the execution time of the same movement.

## Figures and Tables

**Table 1:** Sociodemographic characteristics of the sample.

Variables	Frequency (%)
Gender	
Male	27 (45%)
Female	33 (55%)
*n* (%)	60 (100%)
Age (M ± SD)	83.5 ± 7.80
70–79 years	16 (26.67%)
80–89 years	26 (43.33%)
90–100 years	18 (30%)

*n*, sample size; M, mean; SD, standard deviation.

**Table 2:** Results of the descriptive analysis in each subscale.

Subscale		Mean	CI 95%	SD
IVS	1st S	14.42	13.63–15.21	3.055
	2nd S	16.67	15.86–17.47	3.112
EVS	1st S	18.08	17.32–18.84	2.936
	2nd S	20.58	19.90–21.27	2.651
KS	1st S	12.25	11.59–12.91	2.55
	2nd S	14.25	13.56–14.94	2.678

IVS, Internal Visual Subscale; EVS, External Visual Subscale; KS, Kinesthetic Subscale; 1st S, first session; 2nd S, second session; CI, confidence interval; SD, standard deviation.

**Table 3:** Results of the analysis of internal consistency and test-retest reliability of Movement Imagery Questionnaire-3 (MIQ-3).

N_o_	K_w_		CI (95%)	*p*
Item 1	0.29		0.14–0.45	<0.0001
Item 2	0.47		0.31–0.64	<0.0001
Item 3	0.36		0.20–0.52	<0.0001
Item 4	0.70		0.58–0.82	<0.0001
Item 5	0.26		0.11–0.41	<0.0001
Item 6	0.34		0.18–0.49	<0.0001
Item 7	0.30		0.15–0.45	<0.0001
Item 8	0.71		0.59–0.82	<0.0001
Item 9	0.39		0.24–0.54	<0.0001
Item 10	0.40		0.24–0.55	<0.0001
Item 11	0.25		0.12–0.39	=0.001
Item 12	0.70		0.55–0.81	<0.0001
	**Cronbach’s Alpha**	**ICC**	**CI (95%)**	***p***
IVS	0.615	0.611	0.02–0.83	<0.001
EVS	0.651	0.534	0.07–0.80	<0.001
KS	0.556	0.691	0.07–0.90	<0.001

N_o_, item number in MIQ-3; K_W_, kappa value; CI, confidence interval; *p*, statistical significance; IVS, Internal Visual Subscale; EVS, External Visual Subscale; KS, Kinesthetic Subscale; ICC, intraclass correlation.

**Table 4:** The mixed factorial analysis of variance (ANOVA) results of the differences in motor imagery (MI) ability measured through the MIQ-3, according to sex.

Inter-Subject Factor	MIQ-3			
Sex	Internal Visual Subscale		Mean (SD)	
			First session	Second session
		Male (*n* = 27)	14.41 (2.76)	16.56 (2.91)
		Female (*n* = 33)	14.42 (3.32)	16.76 (3.31)
		Time × sex interaction	F_(1, 58)_ = 0.12; *p* = 0.736; η_p_^2^ = 0.002	
		Inter-subject factor (Sex)	F_(1, 58)_ = 0.02; *p* = 0.886; η_p_^2^ < 0.001	
		Inter-group mean difference and CI (95%)	First session	−0.02 (−1.62; 1.58) *p* = 0.983 d < 0.01
			Second session	−0.20 (−1.84; 1.43) *p* = 0.805 d = 0.06
		Intra-subject factor	F_(1, 58)_ = 67.68; *p* < 0.001; η_p_^2^ = 0.537	
		Intra-group mean difference and CI (95%)	Male	−2.15 (−2.96; −1.34) *p* < 0.001
			Female	−2.33 (−3.07; −1.60) *p* < 0.001
	External Visual Subscale		Mean (SD)	
			First session	Second session
		Male (*n* = 27)	18.41 (2.42)	20.67 (2.24)
		Female (*n* = 33)	17.82 (3.31)	20.52 (2.98)
		Time × sex interaction	F_(1, 58)_ = 0.71; *p* = 0.403; η_p_^2^ = 0.012	
		Inter-subject factor (sex)	F_(1, 58)_ = 0.30; *p* = 0.589; η_p_^2^ = 0.005	
		Inter-group mean difference and CI (95%)	First session	0.59 (−0.94; 2.12) *p* = 0.444 d = 0.20
			Second session	0.15 (−1.24; 1.54) *p* = 0.828 d = 0.06
		Intra-subject factor	F_(1, 58)_ = 91.13; *p* < 0.001; η_p_^2^ = 0.611	
		Intra-group mean difference and CI (95%)	Male	−2.26 (−3.03; −1.49) *p* < 0.001
			Female	−2.70 (−3.39; −2.00) *p* < 0.001
	Kinesthetic Subscale		Mean (SD)	
			First session	Second session
		Male (*n* = 27)	12.48 (2.55)	14.26 (2.68)
		Female (*n* = 33)	12.06 (2.59)	14.24 (2.72)
		Time × sex interaction	F_(1, 58)_ = 1.64; *p* = 0.205; η_p_^2^ = 0.028	
		Inter-subject factor (Sex)	F_(1, 58)_ = 0.11; *p* = 0.743; η_p_^2^ = 0.002	
		Inter-group mean difference and CI (95%)	First session	0.42 (−0.91; 1.75) *p* = 0.530 d = 0.16
			Second session	0.02 (−1.39; 1.42) *p* = 0.981 d < 0.01
		Intra-subject factor	F_(1, 58)_ = 157.80. *p* < 0.001; η_p_^2^ = 0.731	
		Intra-group mean difference and CI (95%)	Male	−1.78 (−2.25; −1.31) *p* < 0.001
			Female	−2.18 (−2.61; −1.76) *p* < 0.001

*n*, sample size; SD, standard deviation; CI, confidence interval; *p*, statistical significance; F, Fisher; η_p_^2^, partial eta-squared coefficient.

**Table 5:** The mixed factorial ANOVA results of differences in MI ability as measured by the MIQ-3, by age range.

Inter-Subject Factor	MIQ-3				
Age range	Internal Visual Subscale		Mean (SD)		
			First session	Second session	
		70–79 years (*n* = 16)	18.12 (2.22)	19.25 (2.02)	
		80–89 years (*n* = 26)	13.77 (1.93)	17.73 (1.43)	
		90–100 years (*n* = 18)	12.06 (1.77)	12.83 (1.86)	
		Time × age range interaction	F_(2, 57)_ = 31.68; *p* < 0.001; η_p_^2^ = 0.526		
		Inter-subject factor (age range)	F_(2, 57)_ = 57.54; *p* < 0.001; η_p_^2^ = 0.669		
		Inter-group mean difference and CI (95%)	First session	70–79 vs. 80–89	4.36 (2.82; 5.89) *p* < 0.001 d = 2.13
				70–79 vs. 90–100	6.07 (4.41; 7.73) *p* < 0.001 d = 3.04
				80–89 vs. 90–100	1.71 (0.23; 3.19) *p* = 0.018 d = 0.92
			Second session	70–79 vs. 80–89	1.52 (0.16; 2.88) *p* = 0.023 d = 0.91
				70–79 vs. 90–100	6.42 (4.95; 7.88) *p* < 0.001 d = 3.32
				80–89 vs. 90–100	4.88 (3.59; 6.21) *p* < 0.001 d = 3.03
		Intra-subject factor	F_(1, 57)_ = 109.86; *p* < 0.001; η_p_^2^ = 0.643		
		Intra-group mean difference and CI (95%)	70–79 years	−1.13 (−1.86; −0.39) *p* = 0.003	
			80–89 years	−3.96 (−4.54; −3.39) *p* < 0.001	
			90–100 years	−0.78 (−1.47; −0.09) *p* = 0.028	
	External Visual Subscale		Mean (SD)		
			First session	Second session	
		70–79 years (*n* = 16)	21.13 (2.03)	23.00 (1.55)	
		80–89 years (*n* = 26)	17.62 (2.32)	21.38 (1.42)	
		90–100 years (*n* = 18)	16.06 (2.24)	17.28 (1.13)	
		Time × age range interaction	F_(2, 57)_ = 14.03; *p* < 0.001; η_p_^2^ = 0.330		
		Inter-subject factor (age range)	F_(2, 57)_ = 45.60; *p* < 0.001; η_p_^2^ = 0.615		
		Inter-group mean difference and CI (95%)	First session	70–79 vs. 80–89	3.51 (1.77; 5.25) *p* < 0.001 d = 1.58
				70–79 vs. 90–100	5.07 (3.19; 6.95) *p* < 0.001 d = 2.36
				80–89 vs. 90–100	1.56 (−0.12; 3.24) *p* = 0.077 d = 0.68
			Second session	70–79 vs. 80–89	1.62 (0.54; 2.69) *p* = 0.001 d = 1.10
				70–79 vs. 90–100	5.72 (4.56; 6.89) *p* < 0.001 d = 4.26
				80–89 vs. 90–100	4.11 (3.07; 5.15) *p* < 0.001 d = 3.13
		Intra-subject factor	F_(1, 57)_ = 109.03; *p* < 0.001; η_p_^2^ = 0.657		
		Intra-group mean difference and CI (95%)	70–79 years	−1.88 (−2.71; −1.04) *p* < 0.001	
			80–89 years	−3.77 (−4.42; −3.12) *p* < 0.001	
			90–100 years	−1.22 (−2.01; −0.44) *p* = 0.003	
	Kinesthetic Subscale		Mean (SD)		
			First session	Second session	
		70–79 years (*n* = 16)	15.44 (1.86)	17.44 (1.63)	
		80–89 years (*n* = 26)	11.62 (1.42)	14.12 (1.28)	
		90–100 years (*n* = 18)	10.33 (1.61)	11.61 (1.79)	
		Time × age range interaction	F_(2, 57)_ = 6.28; *p* = 0.003; η_p_^2^ = 0.181		
		Inter-subject Factor (age range)	F_(2, 57)_ = 60.47; *p* < 0.001; η_p_^2^ = 0.680		
		Inter-group mean difference and CI (95%)	First session	70–79 vs. 80–89	3.82 (2.57; 5.08) *p* < 0.001 d = 2.39
				70–79 vs. 90–100	5.10 (3.75; 5.46) *p* < 0.001 d = 2.95
				80–89 vs. 90–100	1.28 (0.07; 2.05) *p* = 0.034 d = 0.86
			Second session	70–79 vs. 80–89	3.32 (2.12; 4.53) *p* < 0.001 d = 2.34
				70–79 vs. 90–100	5.83 (4.52; 7.13) *p* < 0.001 d = 3.40
				80–89 vs. 90–100	2.50 (1.34; 3.67) *p* < 0.001 d = 1.67
		Intra-subject factor	F_(1, 57)_ = 168.59. *p* < 0.001; η_p_^2^ = 0.747		
		Intra-group mean difference and CI (95%)	70–79 years	−2.00 (−2.56; −1.44) *p* < 0.001	
			80–89 years	−2.50 (−2.94; −2.06) *p* < 0.001	
			90–100 years	−1.28 (−1.81; −0.75) *p* < 0.001	

*n*, sample size; SD, standard deviation; CI, confidence interval; *p*, statistical significance; F, Fisher; η_p_^2^, partial eta-squared coefficient.

**Table 6:** Temporal congruency concerning sex.

Inter-Subject Factor	Temporal Congruence			
Sex		Male (*n* = 27) Median (Q1–Q3)	Female (*n* = 33) Median (Q1–Q3)	Effect Size
	Elbow Flexo-Extension Difference	0.20 (−0.30; 0.35)	0.00 (−0.20; 0.30)	*p* = 0.720 r = 0.05
	Knee Flexo-Extension Difference	0.10 (−0.05; 0.40)	0.10 (−0.30; 0.30)	*p* = 0.162 r = 0.02
	Get up and Sit down Difference	−0.60 (−0.80; −0.25)	−0.60 (−0.80; −0.30)	*p* = 0.905 r = 0.12

Mann–Whitney U-test was used; Q1–Q3, first through third quartiles; r, Rosenthal’s “r”; *p*, statistical significance.

**Table 7:** Temporal congruence concerning age.

Inter-Subject Factor	Temporal Congruence					
Age range		70–79 Years Median (Q1–Q3)	80–89 Years Median (Q1–Q3)	90–100 Years Median (Q1–Q3)	Effect Size	
	Elbow Flexo-Extension Difference	−0.25 (−0.30; −0.15)	0.20 (−0.10; 0.30)	0.20 (−0.10; 0.50)	Global	*p* = 0.001
					70–79 vs. 80–89	*p* = 0.007
					70–79 vs. 90–100	*p* = 0.001
					80–89 vs. 90–100	*p* = 0.999
	Knee Flexo-Extension Difference	−0.05 (−0.40; 0.10)	0.10 (−0.20; 0.40)	0.35 (0.10; 0.50)	Global	*p* = 0.008
					70–79 vs. 80–89	*p* = 0.086
					70–79 vs. 90–100	*p* = 0.007
					80–89 vs. 90–100	*p* = 0.752
	Get up and Sit down Difference	−0.65 (−1.10; −0.20)	−0.45 (−0.80; −0.20)	−0.70 (−0.90; −0.30)	Global	*p* = 0.134
					70–79 vs. 80–89	*p* = 0.312
					70–79 vs. 90–100	*p* = 0.999
					80–89 vs. 90–100	*p* = 0.258

Q1–Q3, first through third quartiles.

## Data Availability

Not applicable.
